# Exogenous Ochronosis Masquerading As Melasma in Skin of Color: Indications for Early Detection and Updated Clinical Review of Diagnosis, Pathomechanism, and Management

**DOI:** 10.7759/cureus.115528

**Published:** 2026-08-31

**Authors:** Beylul Negassi, Celina Sit, Kritin K Verma, Rebecca Vasquez, Seemal Desai, Daniel P Friedmann, Michelle Tarbox

**Affiliations:** 1 Dermatology, School of Medicine, State University of New York (SUNY) Downstate Health Sciences University, Brooklyn, USA; 2 Medicine, Texas Tech University Health Sciences Center El Paso Paul L. Foster School of Medicine, El Paso, USA; 3 Dermatology, School of Medicine, Texas Tech University Health Sciences Center, Lubbock, USA; 4 Dermatology, The University of Texas Southwestern Medical Center, Dallas, USA; 5 Dermatology, Innovative Dermatology, Plano, USA; 6 Dermatology, Westlake Dermatology Clinical Research Center, Westlake Dermatology &amp; Cosmetic Surgery, Austin, USA; 7 Dermatology, Texas Tech University Health Sciences Center, Lubbock, USA

**Keywords:** exogenous ochronosis, fitzpatrick type v, hydroquinone, melasma, skin of color

## Abstract

Exogenous ochronosis (EO) is an uncommon but potentially disfiguring complication of prolonged topical hydroquinone use. It occurs most frequently in individuals with darker skin types and may clinically resemble melasma, often leading to delayed recognition and continued exposure to the offending agent. A 66-year-old female with Fitzpatrick skin type V presented with progressive facial hyperpigmentation, initially diagnosed as melasma and treated with repeated courses of topical hydroquinone. Despite therapy, pigmentation darkened over time, developing a slate-gray to blue-black discoloration. Reevaluation raised concern for EO based on clinical features and paradoxical worsening with continued treatment. Hydroquinone was discontinued, and the patient was treated with topical retinoids and strict photoprotection, resulting in stabilization of pigmentation at follow-up. Exogenous ochronosis remains an underrecognized diagnosis and a common source of iatrogenic progression when misclassified as treatment-resistant melasma. This case highlights a common diagnostic pitfall and provides clinical images and practical guidance for earlier recognition and management of EO in patients with skin of color.

## Introduction

Pigmentary disorders are common in individuals with skin of color and can have a significant psychosocial impact [1]. Melasma, characterized by symmetric hyperpigmented macules and patches on sun-exposed areas, is often treated with depigmenting agents such as topical hydroquinone [2]. Exogenous ochronosis (EO) is a rare but disfiguring complication thought to arise from both prolonged use and higher concentrations of hydroquinone, and it presents as paradoxical blue-gray hyperpigmentation resulting from dermal pigment deposition [2]. Early EO may clinically resemble melasma, leading to diagnostic confusion and continued use of hydroquinone, which can exacerbate disease progression. Despite increasing recognition, EO is still underdiagnosed, particularly in patients with darker skin types [3]. This article provides an updated clinical review of EO and photographic examples that may help clinicians distinguish it from melasma earlier, especially when pigmentation fails to improve with standard therapy.

## Case presentation

A 66-year-old female with Fitzpatrick skin type V presented with a one-year history of worsening facial hyperpigmentation. The patient reported that pigmentation began approximately three years prior as dark brown patches on the cheeks and was diagnosed as melasma by an outside dermatologist (Figure 1). She was started on topical hydroquinone 4% applied twice daily to affected areas. The patient initially noted improvement and continued therapy, later supplementing treatment with additional over-the-counter skin-brightening products. Over the following year, however, the pigmentation gradually darkened rather than lightened. Hydroquinone use continued intermittently for approximately three years in total (Figure 2). On examination, the patient demonstrated symmetric hyperpigmented patches involving the preauricular regions, inferior temples, and malar areas (Figure 3). The pigmentation consisted of confluent brown to blue-gray patches with an irregular, mottled appearance. Areas of darker slate-gray discoloration were interspersed within a background of diffuse hyperpigmentation, producing a reticulated pattern (Figure 4). Close inspection revealed subtle textural changes, including multiple pinpoint to small macular darker foci scattered within the affected areas. The lesions lacked sharply demarcated borders and extended beyond the typical distribution of melasma. No associated erythema, scale, or papular component was observed.

**Figure 1:**
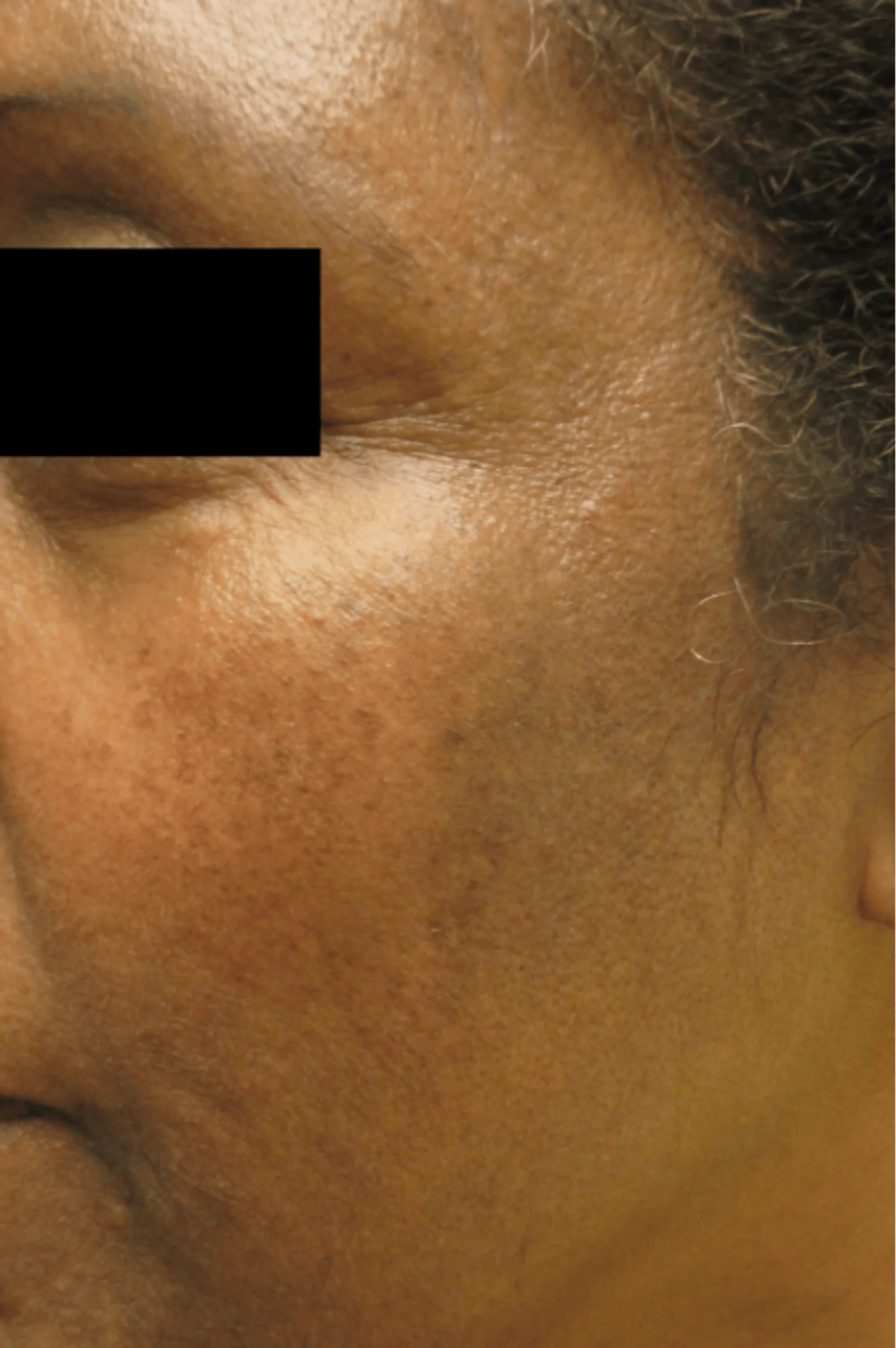
Hyperpigmentation at the time of initial clinical diagnosis of melasma in a patient with Fitzpatrick skin type V

**Figure 2:**
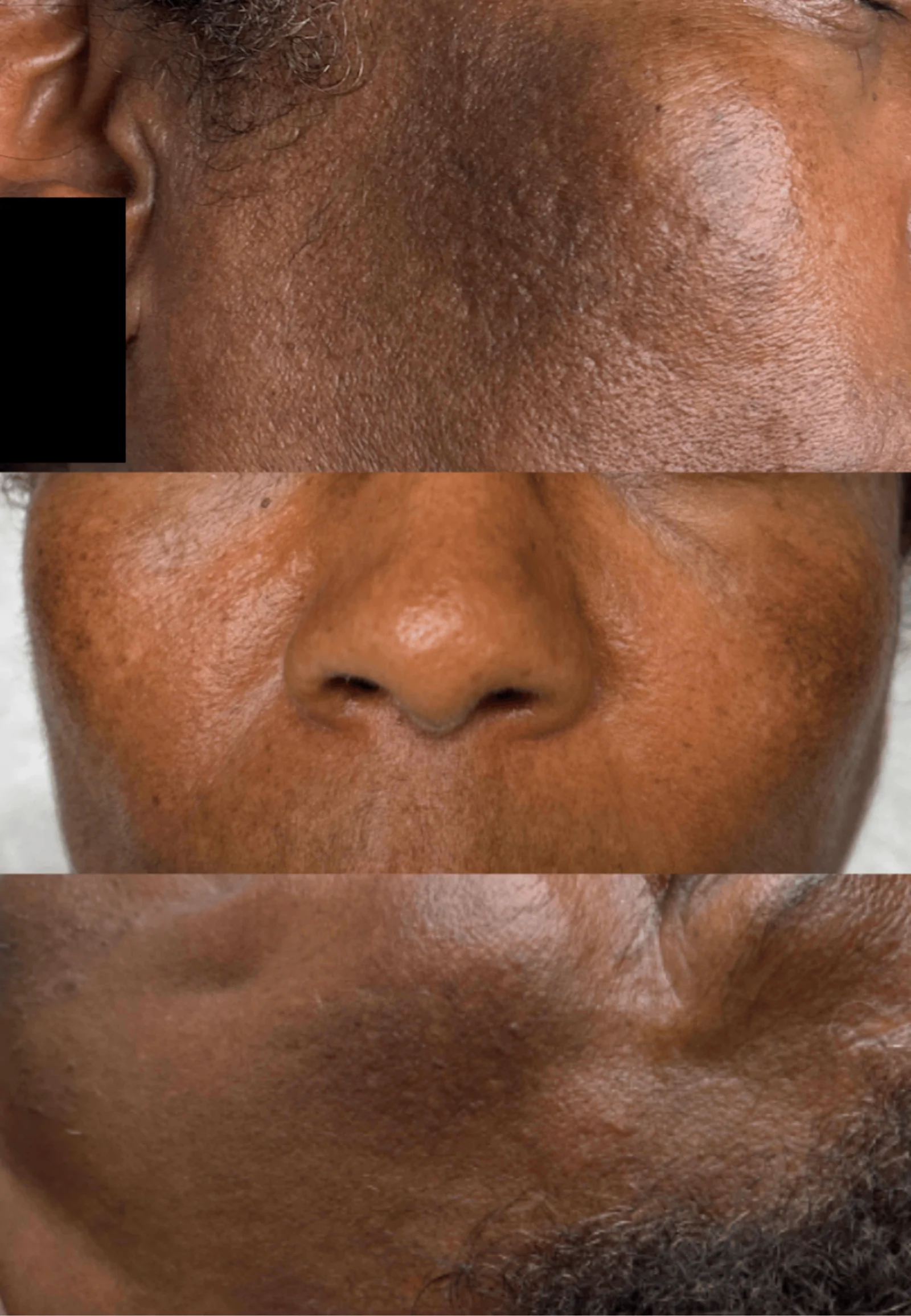
Facial hyperpigmentation after prolonged hydroquinone use Composite clinical image demonstrating progressive facial hyperpigmentation raising concern for EO in a patient with Fitzpatrick skin type V. EO: Exogenous ochronosis

**Figure 3:**
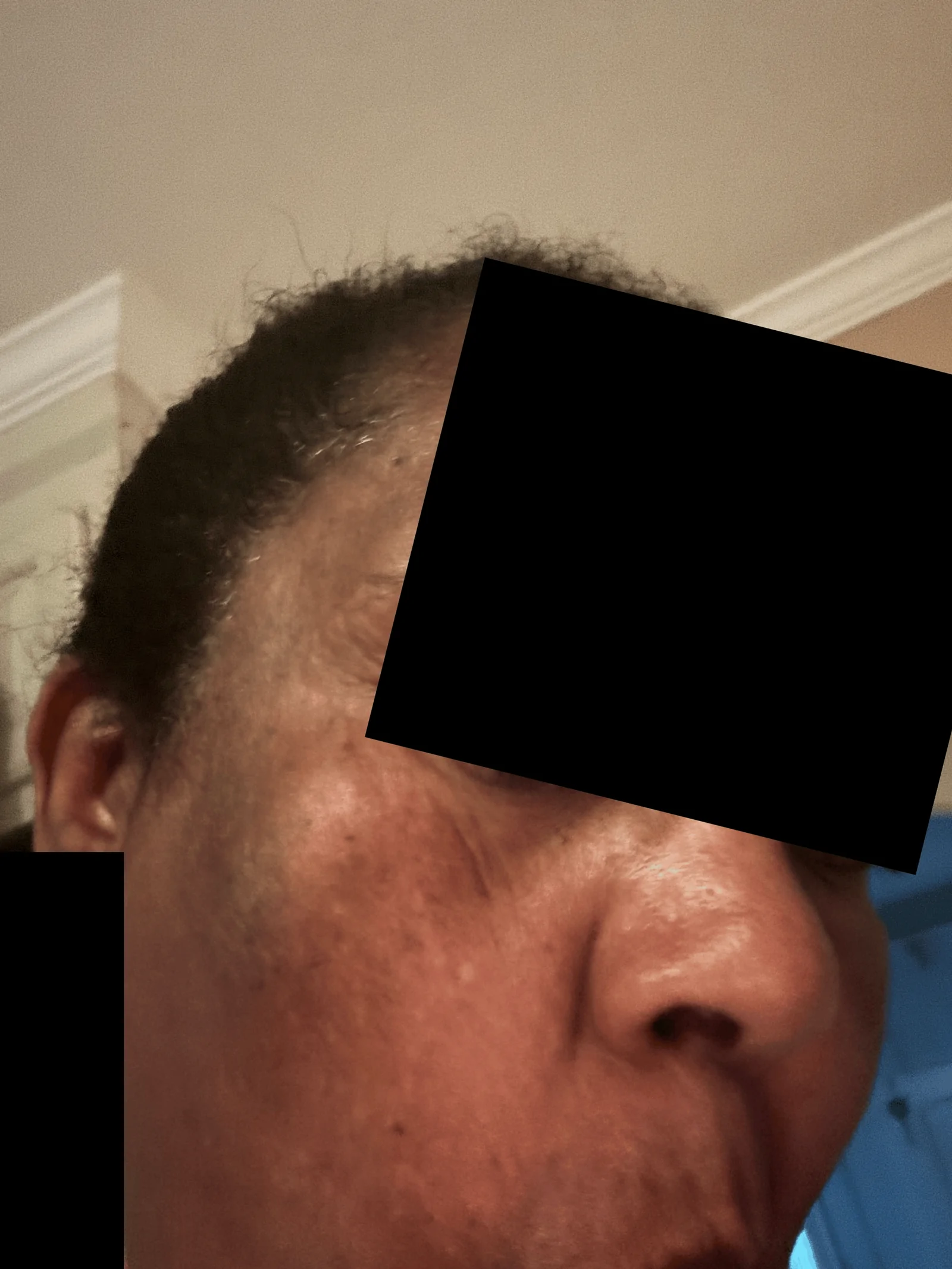
Hyperpigmented patches involving inferior temple and lateral cheek

**Figure 4:**
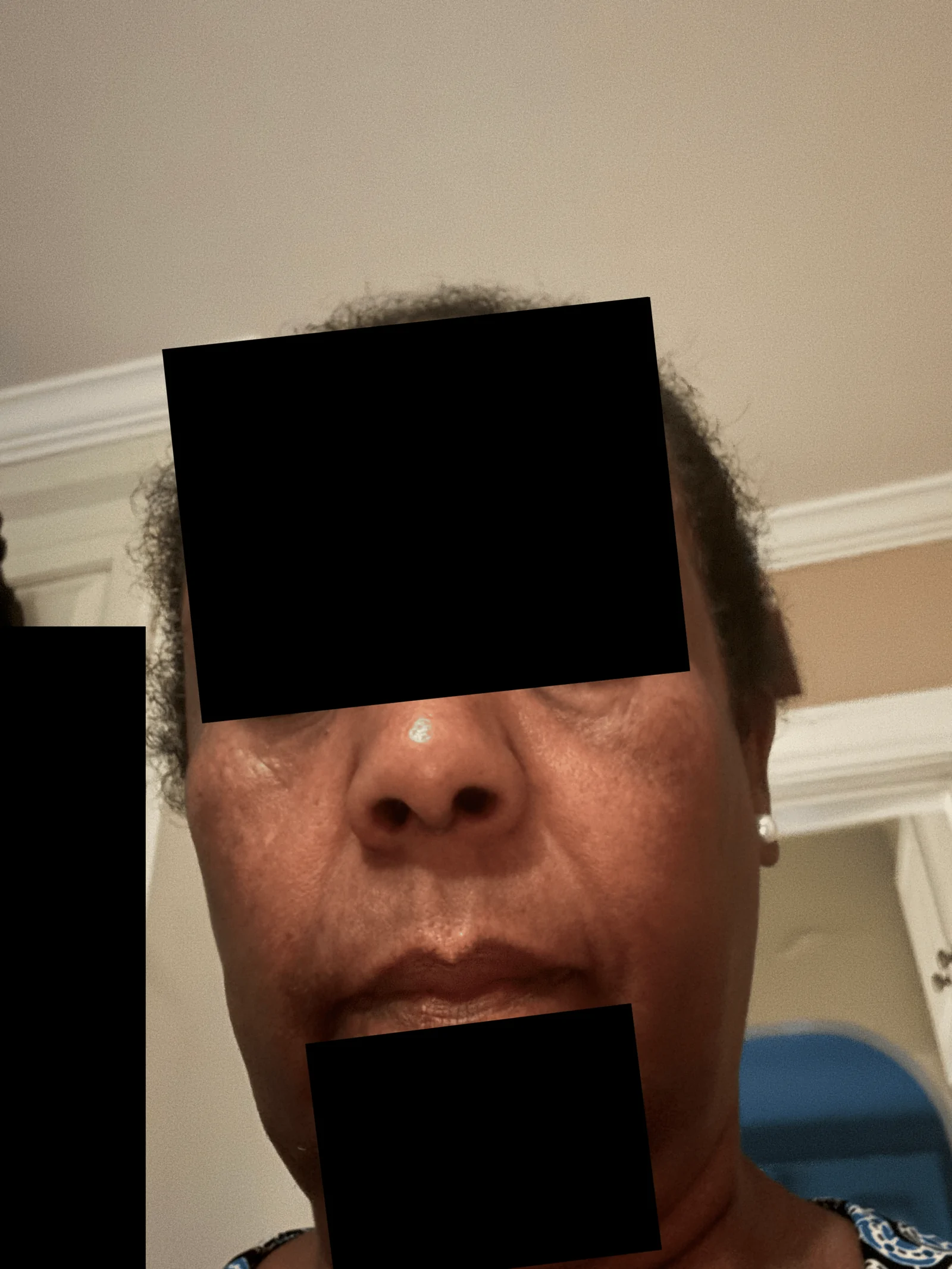
Frontal view of face with hyperpigmented patches on infra-orbital regions

The blue-gray hue and paradoxical progression despite depigmenting therapy were not typical of melasma and prompted reconsideration of the diagnosis. Dermoscopic examination was not documented at the time of evaluation. The patient declined a biopsy of the affected area. Nonetheless, the clinical presentation, history of prolonged hydroquinone use, and worsening of pigmentation raised strong concern for EO. Hydroquinone therapy was discontinued immediately. The patient was counseled regarding the likely chronic course of the condition and advised to maintain strict photoprotection, topical retinoid therapy, and sunscreen use. At the follow-up three months later, pigmentation had stabilized without further progression.

## Discussion

Clinical context and diagnostic pitfall

Exogenous ochronosis is an overlooked complication of hydroquinone use and is frequently misdiagnosed as treatment-resistant melasma, particularly in patients with skin of color. In a systematic review of 126 cases, over half of affected patients had Fitzpatrick skin types V-VI [4]. Since EO arises most often in patients with darker phototypes, subtle early changes may be overlooked against baseline pigmentation, delaying recognition until the condition has progressed significantly [4]. Recent literature has further characterized the clinical spectrum of EO, including risk factors related to prolonged hydroquinone exposure, evolving dermoscopic features, and the impact of diagnostic delay on disease severity [3,4]. This diagnostic overlap contributes to a cycle of treatment escalation and worsening disease. The present case illustrates this common clinical scenario and offers visual references in Fitzpatrick skin type V that may help clinicians recognize EO earlier when treatment response is atypical.

Epidemiology and clinical context

Exogenous ochronosis is most commonly associated with prolonged topical hydroquinone exposure and has been reported with both prescription and over-the-counter formulations, including concentrations as low as 2% [3,4]. Risk increases with higher concentrations (≥4%), prolonged duration of use, occlusive vehicles, and ultraviolet exposure. Diagnostic delay is well documented. In one cohort, approximately one-third of patients with EO were initially diagnosed with melasma, and systematic reviews report median hydroquinone exposure durations of several years, with some cases extending beyond a decade (Table 1) [3-5]. Exogenous ochronosis may also develop in individuals initially diagnosed with true melasma, representing a transition from epidermal melanosis and/or dermal melanophages (in mixed melasma with both epidermal and dermal involvement) to dermal ochronotic deposition that is clinically interpreted as treatment failure. Once established, EO tends to persist and is significantly more resistant to therapy.

**Table 1:** Clinical and diagnostic features distinguishing EO from melasma EO: Exogenous ochronosis

Features	EO	Melasma
History	After months to years of hydroquinone/phenol use [3-6]	Often associated with hormonal triggers (pregnancy, oral contraceptive pills); no hydroquinone link [2,7]
Patient demographic	Typically Fitzpatrick III-VI, middle-aged; individuals with skin of color [3-6]	Fitzpatrick I-IV (more common III-V) [2,7]
Color	Slate-gray, blue-black, dark brown patches [3,4,6]	Light-to-dark brown [2,7]
Distribution	Malar, zygomas, temples, neck/back of hands, symmetric [3-6]	Malar areas, forehead, upper lip [2,7]
Texture	Early: macular lesions; Later stages: fine 'caviar-like' papules may develop [3,5,6]	Purely flat macules [2,7]
Dermoscopy	Coarse dark brown/gray globules and arcs; follicular openings often obscured [6,8]	Fine brown reticular network; normal follicles [2,6]
Reflectance confocal microscopy	Non-refractile banana/ovoid bodies in dermis [6]	Increased basal melanin; no dermal bodies [9]
Histology	Ochre-yellow banana-shaped fibers + dermal elastosis; swollen collagen [3-6]	Normal dermis; increased epidermal melanin only [9]
Treatment response	Unresponsive to hydroquinone; lasers, peels, and dermabrasion may provide limited or variable improvement [3-6]	Better; responds to hydroquinone, trichloroacetic acid peels, tranexamic acid [2,7]
Prognosis	Often permanent pigment; may worsen if hydroquinone continues [3-5]	Typically reversible with treatment and sun protection [2,7]

Clinical differentiation (practical distinctions)

Distinguishing EO from melasma relies on careful history and physical examination as detailed in Table 1. It has been described in three clinical stages: stage one with erythema and mild hyperpigmentation, stage two with hyperpigmentation, black colloid milia, and atrophy, and stage three includes papulo-nodular lesions [3]. A key morphologic feature of late-stage EO is the presence of pinpoint hyperpigmented papules embedded within patches, often described as 'caviar-like,' which are absent in melasma [3]. In this case, the patient demonstrated subtle pinpoint dark foci and a reticulated pattern on close inspection, representing characteristic EO features. The goal of this case is to promote earlier recognition by outlining clinical features and diagnostic clues that should prompt suspicion for EO rather than melasma. Clinically, EO should be suspected when hyperpigmentation worsens during hydroquinone therapy, particularly when a blue-gray hue, speckled texture, or papular features emerge in the setting of prolonged use. Similar cases of EO initially diagnosed as melasma are summarized in Table 2, constructed from a focused literature review. Additional differential diagnoses to consider for facial hyperpigmentation include, but are not limited to, maturational dyschromia, acquired dermal macular hyperpigmentation (including lichen planus pigmentosus or erythema dichromium perstans), lentigines, and acquired bilateral nevus of Ota-like macules [10,11].

**Table 2:** Published cases of EO misdiagnosed as melasma EO: Exogenous ochronosis

Study	Patient	Initial diagnosis	Hydroquinone exposure	Duration	Key clinical features	Biopsy	Outcome
Zawar et al., 2004 [12]	30-year-old female; Fitzpatrick scale not specified	Melasma	Hydroquinone 4%	~3 months total	Blue-black malar pigmentation; paradoxical worsening; sun-exposed distribution	Yes: yellowish-brown (ochre) clumps in papillary dermis; swollen, homogenized collagen	Not specifically reported; literature notes slow resolution after discontinuation
Gandhi et al., 2012 [13]	50-year-old female; Fitzpatrick IV-V	Hyperpigmentation (likely melasma)	Hydroquinone 2% to 5% (long-term use; specifically noted 2%)	~7-8 years	Symmetric dark brown–black facial pigmentation; reticulated pattern; atrophy, telangiectasias	Yes: dermal ochre-colored banana-shaped deposits; collagen homogenization	Not reported (only notes that improvement is slow after stopping and treatment is difficult)
Sunkara et al., 2020 [14]	39-year-old female; Fitzpatrick scale not specified	Hyperpigmentation (likely melasma)	Hydroquinone 4%	~1.5 years	Progressive blue-black facial/neck pigmentation; symmetric distribution; mimicking melasma	No	~50% improvement after four months of treatment (peels, microdermabrasion, sunscreen, kojic acid, glutathione)
Dar et al., 2018 [15]	65-year-old male; Fitzpatrick scale not specified	Likely melasma	Hydroquinone 2% + tretinoin + mometasone	~5 years	Diffuse bluish-brown facial plaques; caviar-like papules; involvement of hydroquinone-exposed areas	Yes: ocher-colored globular deposits in papillary dermis; solar elastosis; positive Masson-Fontana stain; features consistent with EO	Not specifically quantified; patient instructed to stop hydroquinone and treated with topical glycolic acid, antioxidants, vitamins A & E; clinical status otherwise stable
Gil et al., 2010 [6]	63-year-old female; phototype V; Hispanic	Melasma	Hydroquinone 2% to 3% + oxybenzone	Several years	Symmetric speckled gray macules; bluish-gray hue; dermoscopic arciform structures; worsening with treatment	Yes: banana-shaped yellow-brown deposits in papillary and mid dermis; elastic fiber degeneration; foreign body reaction	Mild improvement after intense pulsed light + non-hydroquinone depigmenting agents; pigment deposits persisted
Charlín et al., case #1 [8]	56-year-old female; phototype IV	Melasma	Hydroquinone 2% to 4%	~20 years	Gray-brown macules; caviar-like papules; progressive darkening	Yes: solar elastosis, pigment incontinence, ochre banana-shaped fibers in papillary dermis	Partial improvement with retinoic acid, azelaic acid, kojic acid
Charlín et al., case #2 [8]	44-year-old female; phototype IV	Melasma	Hydroquinone 2% to 5%	~10 years	Bilateral blue-brown facial macules; papular changes; initial improvement then worsening	Yes: classic ochronotic fibers	Not reported (patient lost to follow-up)
Charlín et al., case #3 [8]	56-year-old female; phototype V	Melasma	Hydroquinone up to 6%	~25 years	Diffuse gray-brown pigmentation; papules (2 mm to 3 mm); confetti-like depigmentation	Yes: classic ochronosis histology	No improvement with Q-switched Nd:YAG laser
Charlín et al., case #4 [8]	51F; phototype V	Melasma	Hydroquinone up to 5%	~10 years	Brown macules; gray-brown areas; caviar-like papules	Yes: classic ochronosis histology	Retinoic acid + azelaic acid prescribed; no satisfactory improvement
Lazar et al., 2023 (case series, n=25) [3]	Mean age 55; 22 females and three males; Fitzpatrick IV-VI	Frequently misdiagnosed as melasma	≥2% hydroquinone (variable)	Mean 9.2 years (range ~1.5-20 years)	Blue-black/gray facial hyperpigmentation; paradoxical worsening; multifocal involvement	>50% biopsy-confirmed EO	Topicals largely ineffective; best responses seen with Q-switched Alexandrite laser and microneedling

Diagnostic evaluation

Skin biopsy is the diagnostic gold standard, demonstrating characteristic ochre-colored, banana-shaped deposits along collagen and elastic fibers in the papillary dermis [5]. However, in clinical practice, diagnosis often relies on recognition of characteristic features, particularly when biopsy is declined, as with this case. Dermoscopy can be helpful in these cases. As noted in the case presentation, a limitation of this report is the unavailability of dermoscopy at the time of evaluation. Exogenous ochronosis typically reveals coarse brown-gray globules and arciform structures with partial or complete obliteration of follicular openings, whereas melasma demonstrates a fine reticular network with preserved follicular ostia [6]. Reflectance confocal microscopy may demonstrate non-refractile dermal structures corresponding to ochronotic fibers, in contrast to the epidermal melanin changes seen in melasma (Table 1) [6]. Wood’s lamp examination is less reliable, as dermal pigment in EO does not accentuate, and melasma can have dermal, epidermal, or mixed pigmentary patterns [2,5].

Pathophysiology

Melasma and EO represent distinct pathophysiologic processes. Melasma is characterized by dysregulated melanogenesis driven by ultraviolet and visible light exposure, hormonal influences, genetic susceptibility, and inflammatory mediators [7]. In contrast, EO results from dermal deposition of ochronotic pigment following exposure to phenolic compounds such as hydroquinone [16]. Traditionally, proposed mechanisms include inhibition of homogentisate dioxygenase leading to oxidative polymerization of metabolites that bind dermal connective tissue [16]. Recent in vitro evidence has challenged the initial steps of this model. Instead, a study proposed that tyrosinase-catalyzed oxidation of hydroquinone generates low-molecular-weight derivatives that diffuse into the dermis, which initiates ochronotic pigment formation [16]. In both cases, ultraviolet radiation may further promote pigment formation and deposition. Because EO involves structurally integrated dermal pigment, it is substantially less responsive to conventional depigmenting therapies than melasma [5].

Hydroquinone safety and alternative treatments

Hydroquinone remains an effective depigmenting agent when used appropriately but requires careful monitoring [7]. Current clinical guidance emphasizes limiting continuous use to defined treatment courses and reassessing response regularly. Lack of improvement within approximately eight to 12 weeks should prompt reconsideration of the diagnosis [7].

Therapy should be discontinued immediately if paradoxical darkening, new papular changes, or gray-blue discoloration develops [3,4]. Once EO is suspected or confirmed, all hydroquinone-containing products and related phenolic agents should be permanently discontinued [3]. Early cessation at the first signs of adverse change is critical to limit progression. Preventive strategies include using the lowest effective concentration, avoiding prolonged uninterrupted therapy, incorporating treatment breaks, and emphasizing strict photoprotection. A recent retrospective study in the U.S. found that all patients who developed EO in the cohort had obtained their hydroquinone products outside the supervision of a U.S. medical doctor [3]. As a result, many EO cases were associated with over-the-counter products or prescription formulations acquired in other countries. Increased accessibility to hydroquinone, particularly when paired with unsupervised or prolonged use and inadequate sun protection, may increase the risk of EO development [3]. 

Alternatives, including tranexamic acid (TXA), are increasingly recommended for melasma management [17]. Tranexamic acid has demonstrated strong efficacy and safety in multiple studies of melasma. However, clinical trials are needed to confirm that it does not worsen existing ochronotic pigmentation. Such studies could support TXA as a potential alternative to hydroquinone in melasma management, particularly in patients with higher Fitzpatrick skin types, as seen in this case. Other treatment options for melasma without known association with EO include azelaic acid, kojic acid, chemical peels, microneedling, and lasers [18].

Diagnostic challenges

Several factors contribute to frequent misdiagnosis. Exogenous ochronosis and melasma both affect sun-exposed facial areas and are more common in individuals with darker skin types [1]. Early EO may lack distinctive features, leading clinicians to intensify treatment rather than reassess the diagnosis [3].

Over-the-counter availability of hydroquinone, uncertain product concentrations, and use of imported formulations complicate exposure history [4]. In one study, 68% to 84% of skin-lightening creams obtained from West Africa and ethnic shops in Canada contained undeclared ingredients [19]. Even declared ingredients often exceeded U.S. FDA regulatory guidelines for hydroquinone and other harmful agents [19]. Exogenous ochronosis has also been associated with phenolic compounds beyond hydroquinone, as well as the polyphenolic compound epigallocatechin gallate [20]. Clinicians should obtain comprehensive product histories and screen for all skin-lightening products, supplements, and phenolic exposures when evaluating patients with facial hyperpigmentation. Cosmetic camouflage may obscure papular changes, and patients often present late after years of self-treatment. Additionally, EO and melasma may coexist, further complicating interpretation of treatment response [3].

Clinical course and outcomes

Exogenous ochronosis is associated with persistent and often irreversible pigmentation [5]. Even after discontinuation of hydroquinone, improvement is variable and frequently incomplete. Therapeutic options, including topical retinoids, chemical peels, microneedling, dermabrasion, and laser therapies, demonstrate inconsistent efficacy, and outcomes are often limited by the dermal location of pigment (Table 1) [5].

In contrast, melasma is generally more responsive to medical therapy and photoprotection and may improve substantially over time [7]. This distinction emphasizes the importance of early recognition and appropriate management. The patient’s stabilization at the three-month follow-up supports that hydroquinone discontinuation prevents progression even with prolonged exposure.

## Conclusions

Exogenous ochronosis is an underrecognized but clinically significant complication of hydroquinone use that often mimics treatment-resistant melasma. Delayed recognition can lead to continued exposure to the offending agent and irreversible pigmentary change. Clinicians should maintain a high index of suspicion and reassess the diagnosis when pigmentation worsens despite therapy, particularly in patients with skin of color. Early identification and discontinuation of hydroquinone are essential to prevent iatrogenic progression and optimize patient outcomes. Alternative melasma treatments such as TXA should also be considered to prevent this complication, with further studies needed to establish safety and efficacy in patients with EO.
